# Genetic Diversity and Selection Signatures of Two North American Corriente Cattle Herds in Northeastern New Mexico, United States

**DOI:** 10.3390/ijms27187982

**Published:** 2026-09-08

**Authors:** Maximiliano J. Spetter, Richard E. Estell, Eileen M. Armstrong, Craig A. Gifford, Felipe A. Rodríguez Almeida, Eugenio Jara, Andrew Cox, Pablo J. Ross, Andrés R. Perea, Micah Funk, Andrés F. Cibils, Santiago A. Utsumi

**Affiliations:** 1 Department of Animal and Range Sciences, New Mexico State University, Las Cruces, NM 88003, USA; 2 Jornada Experimental Range, Agriculture Research Service, United States Department of Agriculture, Las Cruces, NM 88003, USA; 3 Unidad Mejora Animal, Departamento de Producción Animal, Facultad de Veterinaria, Universidad de la República, Montevideo 11200, Uruguay; 4 Facultad de Zootecnia y Ecología, Universidad Autónoma de Chihuahua, Chihuahua 31000, Mexico; 5 Inguran LLC Dba STgenetics, Navasota, TX 77868, USA; 6 USDA Southern Plains Climate Hub, Oklahoma and Central Plains Agricultural Research Center, El Reno, OK 73036, USA

**Keywords:** Criollo cattle, heritage genetics, Corriente cattle, single nucleotide polymorphisms

## Abstract

The North American Corriente (NAC) is a Criollo-derived cattle breed raised primarily in the Southwestern and Great Plains regions of the United States for rodeo sports and beef production. Despite the substantial practical knowledge surrounding the breed, available information on the genomic makeup of NAC cattle remains limited. To address this gap, a comprehensive genomic analysis of two commercial NAC herds in northeastern New Mexico, United States, was conducted using a ~64 K SNP microarray. A total of 97 NAC cattle were initially sampled; after quality control, relatedness filtering, and population structure analysis, 52 animals were retained for downstream analysis. Population structure and admixture analyses point to a certain degree of genetic differentiation in the NAC population, which could be consistent with a distinct Criollo cattle lineage. This interpretation remains preliminary, however, and would benefit from additional sampling across independent and geographically representative herds. The overlap between functional enrichment results and known quantitative trait loci is consistent with the possibility that some genomic regions associated with movement, fertility, immune response, and carcass and milk traits may have been subject to selection. While informal, anecdotal observations of some of these phenotypic traits have been noted, further research with larger sample sizes and functional validation would be needed before drawing firmer conclusions. This study supports ongoing efforts to genetically characterize, promote, and conserve Criollo cattle populations across the Americas.

## 1. Introduction

Cattle were first introduced to the Americas from the Iberian Peninsula in the late 15th century, with subsequent admixture from African, European, and Zebu cattle over the following centuries [1,2,3,4,5]. These successive introductions shaped the complex genetic structure of their descendants, today known as Criollo or Creole cattle, which have since spread throughout the Americas and adapted to a wide range of climatic and environmental conditions [1,2,6,7,8,9]. This adaptability makes Criollo cattle a valuable genetic resource for improving resilience and sustainability in livestock production, particularly in harsh or highly variable environments.

The North American Corriente (NAC) is one of several Criollo-derived breeds in the Americas. Although Criollo cattle were nearly eliminated in North America by the late 1800s, the modern NAC population likely descends from herds reintroduced from remote regions of Mexico and Central America [1,10,11]. The North American Corriente Association (NACA), founded in 1982, established the NAC designation and now serves as the official breed registry in the United States and Canada [11]. NAC cattle are small-framed, agile, and highly athletic, with prominent horns and a docile temperament, making them well-suited for rodeo sports such as bulldogging and roping, as well as for grass-fed beef production [11,12]. Registered herds span environments ranging from the Canadian plains to the deserts of Arizona and the humid Southeast, underscoring the breed’s broad environmental adaptability.

Despite this adaptability and their cultural and economic importance to regional beef production systems, NAC cattle remain classified as a threatened breed, with a global population estimated at fewer than 5000 individuals [13]. However, genetic diversity, population structure, and admixture patterns of NAC cattle remain insufficiently studied, leaving a critical gap in the genomic knowledge needed to guide conservation and management of this breed.

To help address this gap, the objectives of this study were to (1) assess the genetic diversity and population structure of two NAC herds in northeastern New Mexico, United States, using SNP genotyping data; (2) characterize admixture patterns among these herds and relative to other reference populations; and (3) identify signatures of selection within the NAC population. These findings are expected to contribute to ongoing efforts toward the genetic characterization, promotion, and conservation of Criollo cattle populations in the Americas.

## 2. Results

### 2.1. Genotyping and Quality Control

Ninety-seven ear notch tissue samples were collected from two NAC herds in northeastern New Mexico, United States. One herd was in Roy, Harding County (*n* = 47), whereas the other herd was in Tucumcari, Quay County (*n* = 50). No animals were removed during quality control filtering; however, ten individuals from the Roy herd and seven from the Tucumcari herd were excluded due to close genetic relatedness among animals, leaving 80 individuals (37 from the Roy herd and 43 from the Tucumcari herd) and 56,603 SNPs.

### 2.2. Genetic Structure of North American Corriente Cattle Herds

**Figure 1:**
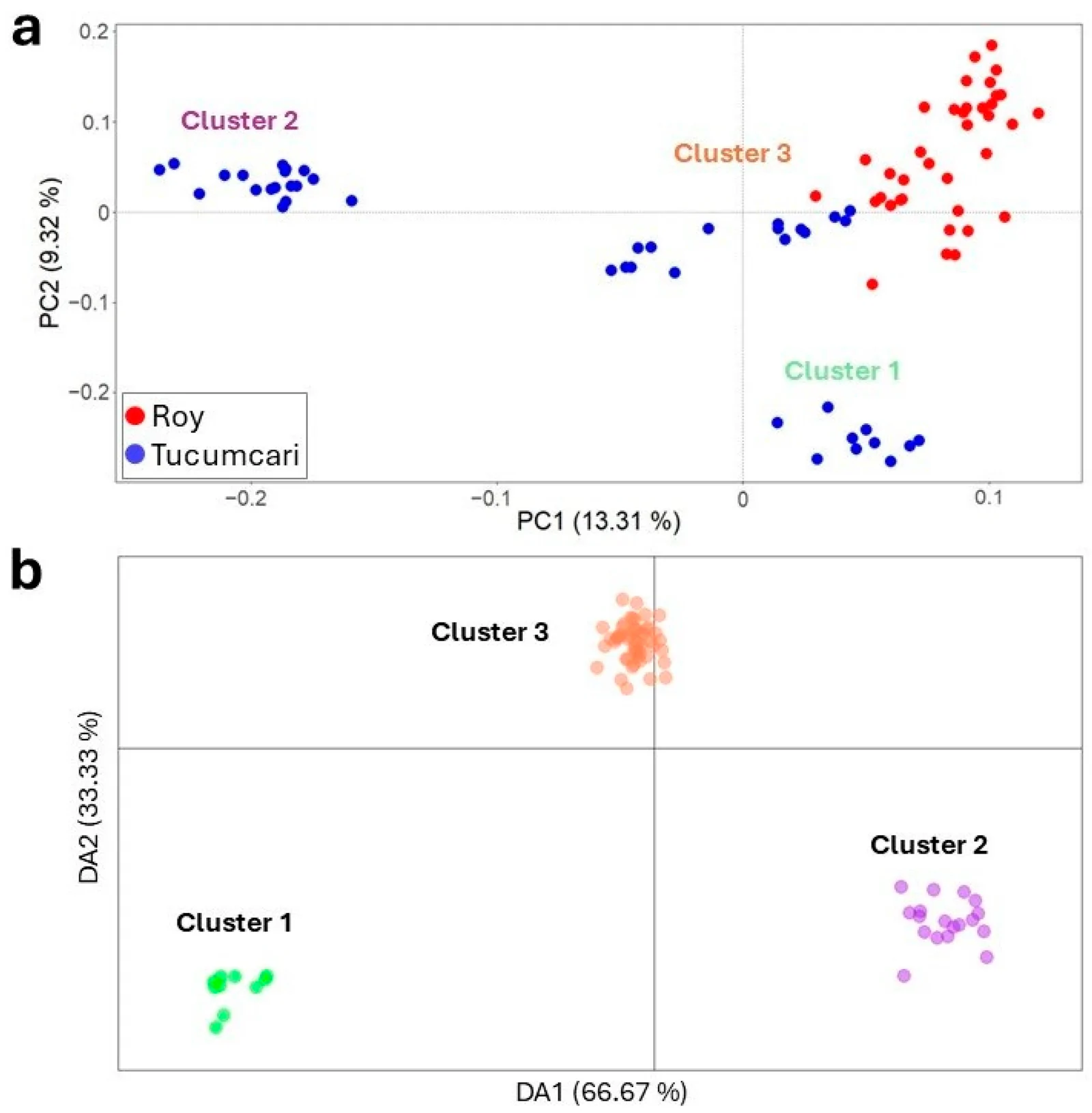
Genetic structure of North American Corriente cattle herds. (**a**) Principal Components Analysis scatter plot. Dots represent individuals and the herds are presented in different colors. (**b**) Discriminant Analysis of Principal Components scatter plot. Twenty-five Principal Components (PC, Figure S1b) and two Discriminant Axes (DA, Figure S1c) were retained. Dots represent individuals and the clusters are presented in different colors.

Genetic structure of the herds was explored using Principal Component Analysis (PCA) and Discriminant Analysis of Principal Components (DAPC). The PCA suggested the possible presence of more than two clusters, despite samples originating from only two herds (Figure 1a); therefore, a DAPC (Figure 1b) was performed to confirm the PCA results. The optimal number of clusters, as determined by the Bayesian Information Criterion (BIC) value, was three (Figure S1a). Cluster 1 (*n* = 10) and Cluster 2 (*n* = 18) consisted solely of animals from the Tucumcari herd, while Cluster 3 (*n* = 52) contained all the individuals from the Roy herd plus 15 from the Tucumcari herd (Figure 1b). The limited and uneven sample sizes of the clusters precluded individual cluster analysis, since simply aggregating all individuals into a single dataset could inflate or mask estimates of genetic diversity, linkage disequilibrium structure, and selection signatures. Therefore, only Cluster 3, which was considered to have a reasonable sample size for independent analysis, was used for downstream analyses.

### 2.3. Genetic Diversity of North American Corriente Cattle Population

The genetic diversity of the NAC population was assessed using multiple parameters. The observed heterozygosity (H_O_, 0.352 ± 0.025) and expected heterozygosity (H_E_, 0.35 ± 0.001) were similar. The inbreeding coefficient based on runs of homozygosity (F_ROH_) per animal ranged from 0.043 to 0.322, with a mean of 0.141. The majority of runs of homozygosity (ROH) segments were short, falling into the >0–2 Mb (58.34%) and >2–4 Mb (18.98%) length classes. Longer ROH segments were less frequent: >4–8 Mb (12.63%), >8–16 Mb (7.58%), and >16 Mb (2.49%).

The historical effective population size (Ne) showed a steady decline until approximately 23 generations ago, followed by a sharp decrease until 16 generations ago. Subsequently, Ne stabilized, reaching an estimated size of 76 in the most recent generation (Figure S2).

### 2.4. Genetic Admixture in the North American Corriente Cattle Population and Their Relationship to Other Cattle Breeds

**Figure 2:**
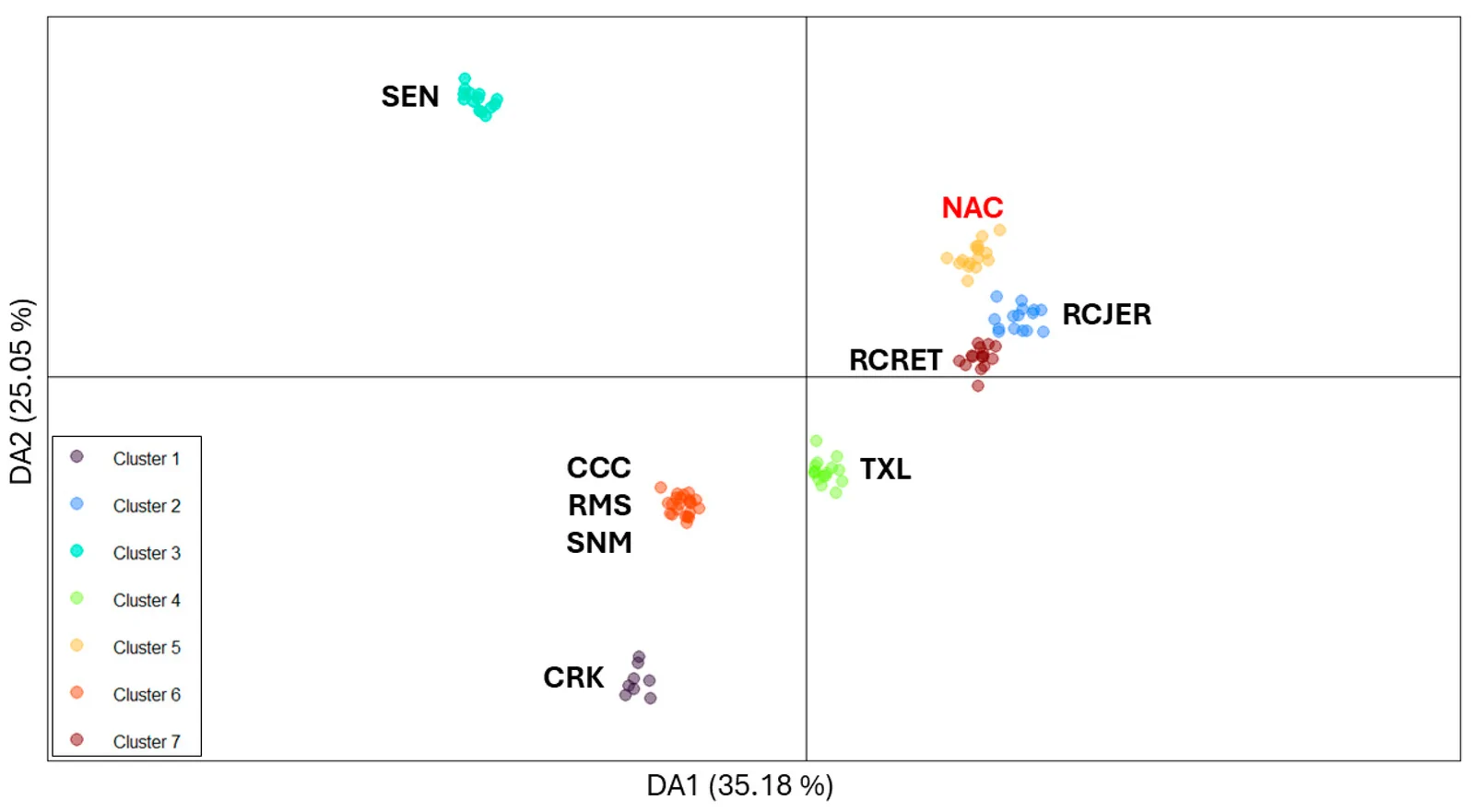
Discriminant Analysis of Principal Components scatter plot of Criollo cattle populations. Dots represent individuals and the clusters are presented in different colors. Thirty-five Principal Components (Figure S3b) and six Discriminant Axes (DA, Figure S3c) were retained. NAC: North American Corriente, CCC: Costeño con Cuernos, CRK: Florida Cracker, RCJER: Rarámuri Criollo cattle from Jornada Experimental Range, RCRET: Rarámuri Criollo cattle from Rancho Experimental Teseachi, SEN: Senepol, RMS: Romosinuano, SNM: San Martinero, TXL: Texas Longhorn.

The NAC population was characterized in relation to other cattle breeds using DAPC and ADMIXTURE. The optimal value of clusters identified by DAPC was seven (K = 1–20; Figure S3a). The DAPC results were consistent with genetic differentiation among the Criollo breeds, pointing to relatively limited admixture and a degree of population structure, except for Cluster 6, which comprised the three Colombian Criollo breeds: Costeño con Cuernos (CCC), Romosinuano (RMS), and San Martinero (SNM) (Figure 2). The NAC was separated from all other clusters along both Discriminant Axes (DA). Along DA1, NAC was more closely related to Rarámuri Criollo (RC) from USDA-ARS Jornada Experimental Range (RCJER), RC from Rancho Experimental Teseachi (RCRET), and Texas Longhorn (TXL), suggesting some shared ancestry or population history. Along DA2, NAC separated from the other three North American Criollo clusters, reflecting greater genetic divergence. The NAC was most differentiated from CCC, RMS, SNM, Florida Cracker (CRK), and Senepol (SEN) breeds, which occupied the opposite side of the DA1 (Figure 2).

**Figure 3:**
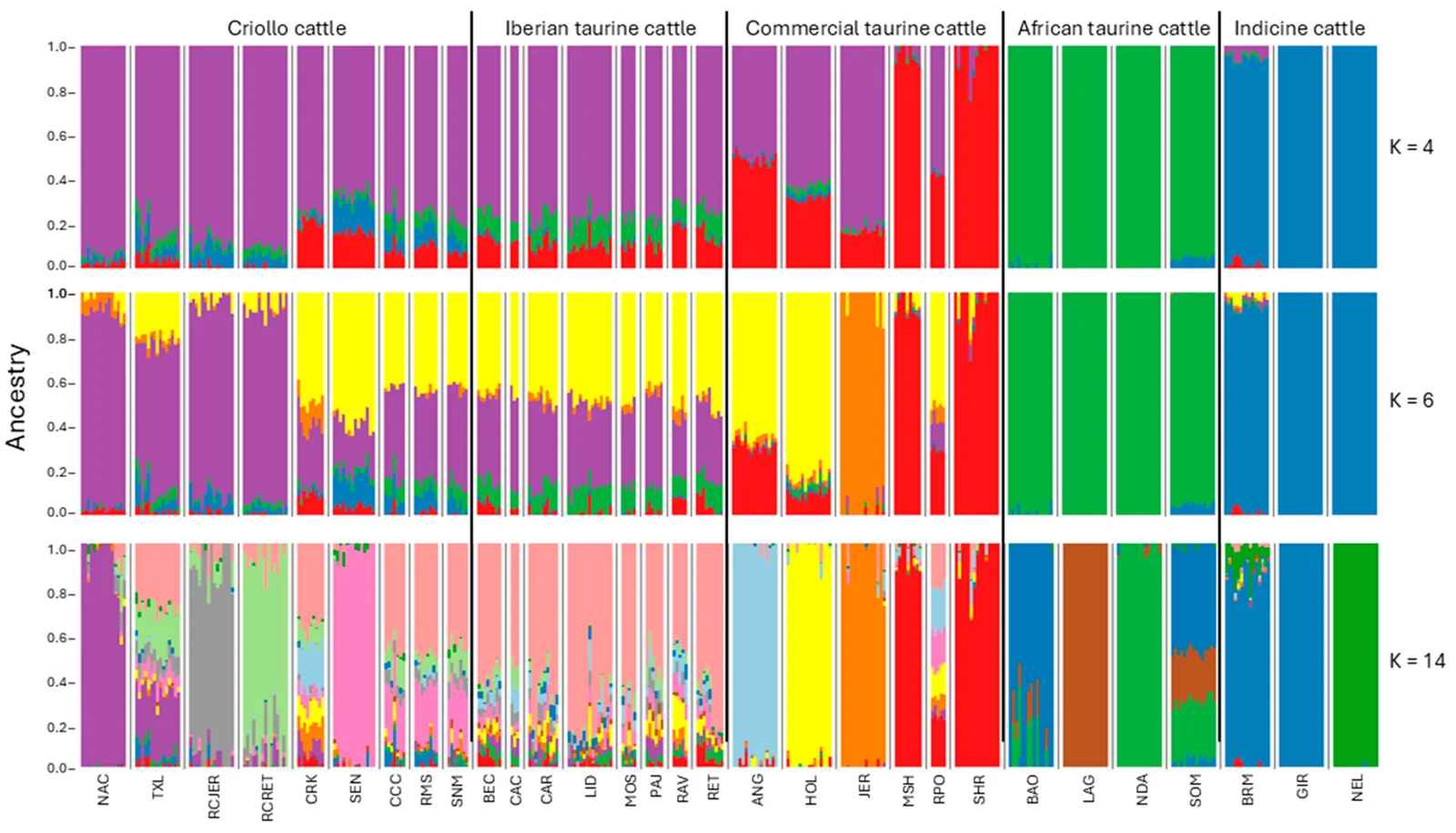
Admixture analysis of North American Corriente cattle along with Criollo, taurine and Indicine cattle breeds inferred using ADMIXTURE v1.4 software. Results for ancestral contributions K = 4, 6, and 14 are shown. The full set of results (K = 2–20) is reported in Figure S4. Each vertical bar represents an individual, and colors within each bar indicate the proportion of ancestry assigned to each inferred genetic cluster. Breeds are grouped by category (Criollo cattle, Iberian taurine cattle, commercial taurine cattle, African taurine cattle, and Indicine cattle) and separated by black vertical lines. Breed abbreviations are shown along the x-axis (NAC: North American Corriente, TXL: Texas Longhorn, RCJER: Rarámuri Criollo cattle from Jornada Experimental Range, RCRET: Rarámuri Criollo cattle from Rancho Experimental Teseachi, CRK: Florida Cracker, SEN: Senepol, CCC: Costeño con Cuernos, RMS: Romosinuano, SNM: San Martinero, BEC: Berrenda en Colorado, CAC: Cachena, CAR: Cardena Andaluza, LID: Lidia, MOS: Mostrenca, PAJ: Pajuna, RAV: Asturiana de los Valles, RET: Retinta, ANG: Angus, HOL: Holstein, JER: Jersey, MSH: Milking Shorthorn, RPO: Red Poll, SHR: Beef Shorthorn, BAO: Baoule, LAG: Lagune, NDA: N’Dama, SOM: Somba, BRM: Brahman, GIR: Gir, NEL: Nelore).

The genetic ancestry of NAC was modeled from K = 2 to K = 20 (Figure S4) using the ADMIXTURE v1.4 software. Representative K values (4, 6, and 14) are described (Figure 3). At K = 4, NAC appeared to show a relatively homogeneous ancestry pattern, with the largest proportion attributed to Iberian taurine, along with smaller contributions from commercial taurine, African taurine, and Indicine groups. This ancestry profile appeared broadly similar to that of the Criollo breeds. At K = 6, the NAC population began to form a partially distinct cluster, yet shared considerable genetic similarity with RCJER, RCRET, and TXL. At K = 14, which showed the lowest cross-validation error (Figure S5), NAC formed a more clearly separated cluster relative to the other Criollo breeds, including TXL, RCJER, and RCRET.

### 2.5. Selection Signature Analysis in North American Corriente Cattle Population

**Figure 4:**
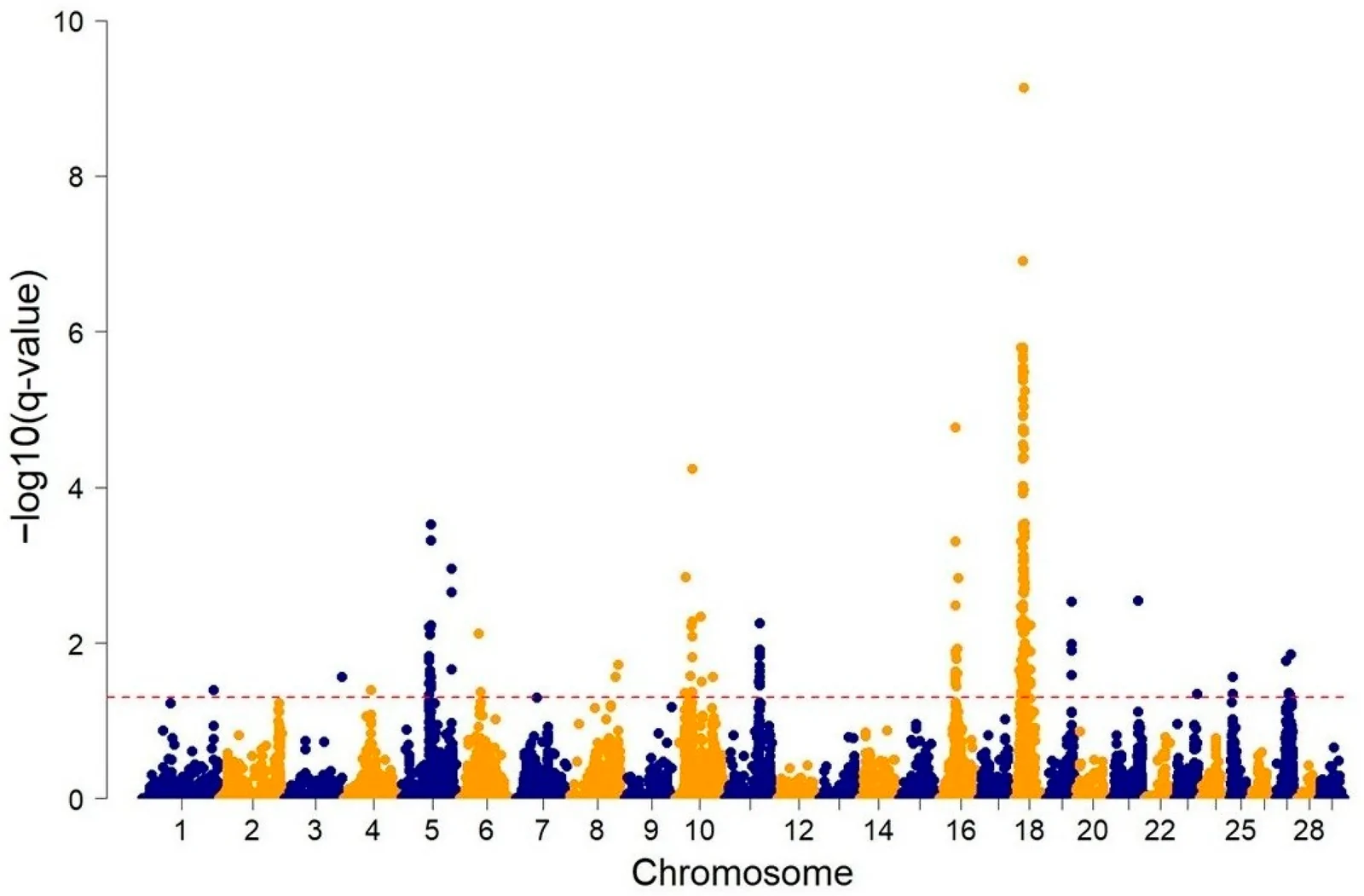
Manhattan plot of decorrelated composite of multiple signal q-values in the North American Corriente cattle population. The red line indicates the threshold of significance at q-value < 0.05 (i.e., −log10(q-value) > 1.3).

A total of 220 SNPs were identified under putative selection across 16 chromosomes (1, 3, 4, 5, 6, 7, 8, 10, 11, 16, 18, 19, 21, 23, 25, and 27) by integrating three complementary intra-population statistics—Tajima’s D (based on site frequency), ROH (based on reduced local variability), and integrated haplotype score (iHS, based on linkage disequilibrium)—within a decorrelated composite of multiple signals (DCMS) framework (Figure 4; Table S2). Chromosome 18 contained the highest number of significant SNPs (*n* = 125), followed by Chromosomes 5 (*n* = 21), 10 (*n* = 16), and 16 (*n* = 16).

### 2.6. Candidate Genes and Quantitative Trait Loci in North American Corriente Cattle Population

A total of 291 genes, associated with a diverse range of traits, were annotated within the significant regions (Table S3). To characterize the biological significance of the genes identified, functional enrichment analyses were performed at both the gene ontology/pathway level and the quantitative trait locus (QTL) level.

**Figure 5:**
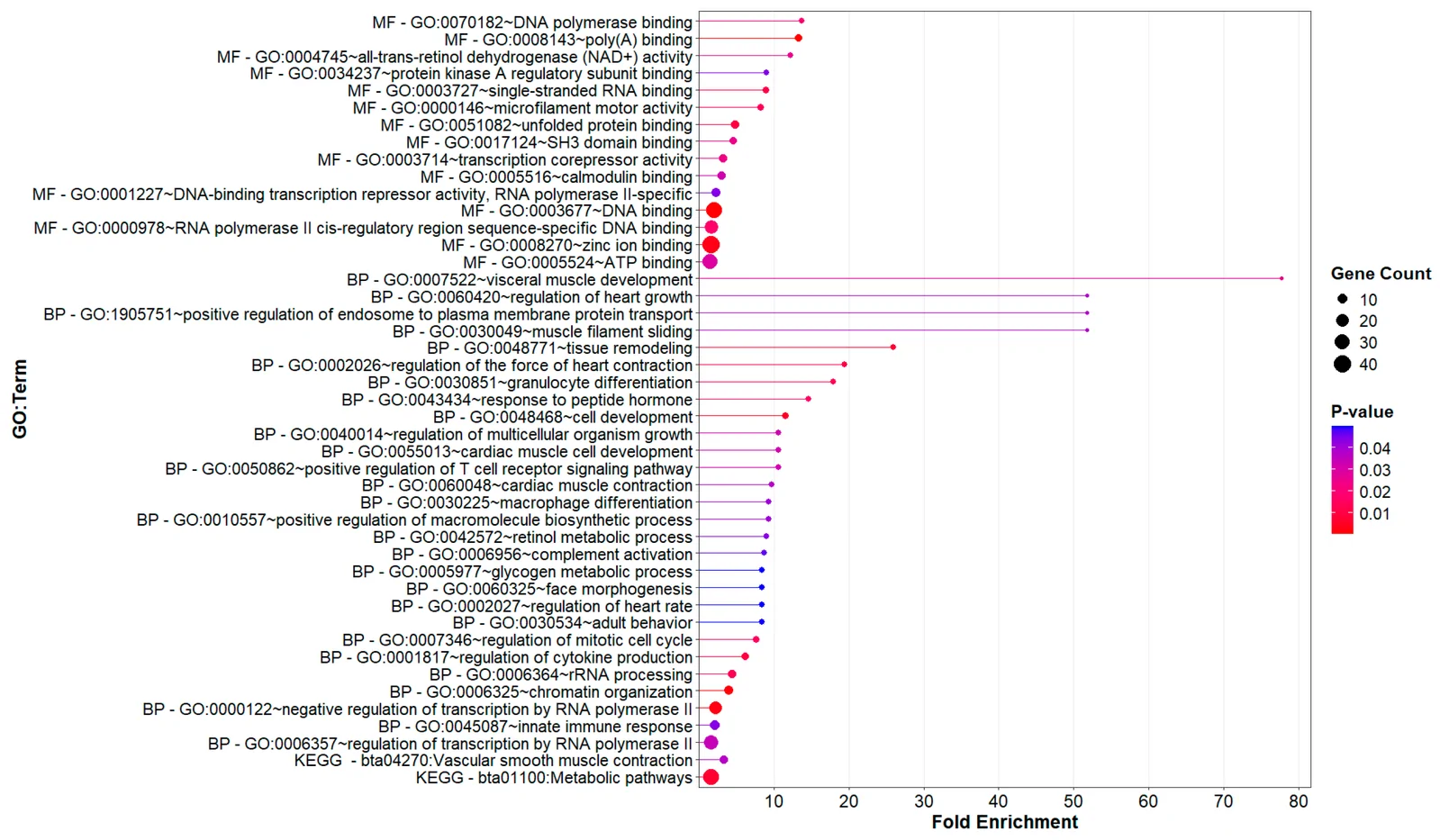
Functional enrichment analysis of candidate genes identified in the North American Corriente cattle population. Terms are grouped by category: molecular function (MF), biological process (BP), and Kyoto Encyclopedia of Genes and Genomes pathway (KEGG). For each term, the x-axis shows Fold Enrichment, defined as the ratio between the observed proportion of candidate genes annotated with the term and the proportion expected based on the background genome. Dot size represents the number of candidate genes annotated with each term (Gene Count). Dot color indicates the *p*-value, with red denoting greater significance.

Gene Ontology (GO) and Kyoto Encyclopedia of Genes and Genomes (KEGG) pathway enrichment analysis identified 45 significantly enriched terms (*p*-value < 0.05; Figure 5). Within the molecular function category, genes under selection were predominantly associated with nucleotide and nucleic acid binding activities, including zinc ion binding, ATP binding, and DNA binding, consistent with broad regulatory and metabolic roles. Among biological process terms, the strongest fold enrichments corresponded to muscle and cardiac development, including visceral muscle development, regulation of heart growth, muscle filament sliding, and regulation of the force of heart contraction, alongside terms related to heart rate regulation and tissue remodeling. Immune-related terms, such as granulocyte and macrophage differentiation, complement activation, innate immune response, and regulation of cytokine production, were also significantly enriched. KEGG pathway analysis supported these findings, identifying vascular smooth muscle contraction and general metabolic pathways as significantly enriched.

**Figure 6:**
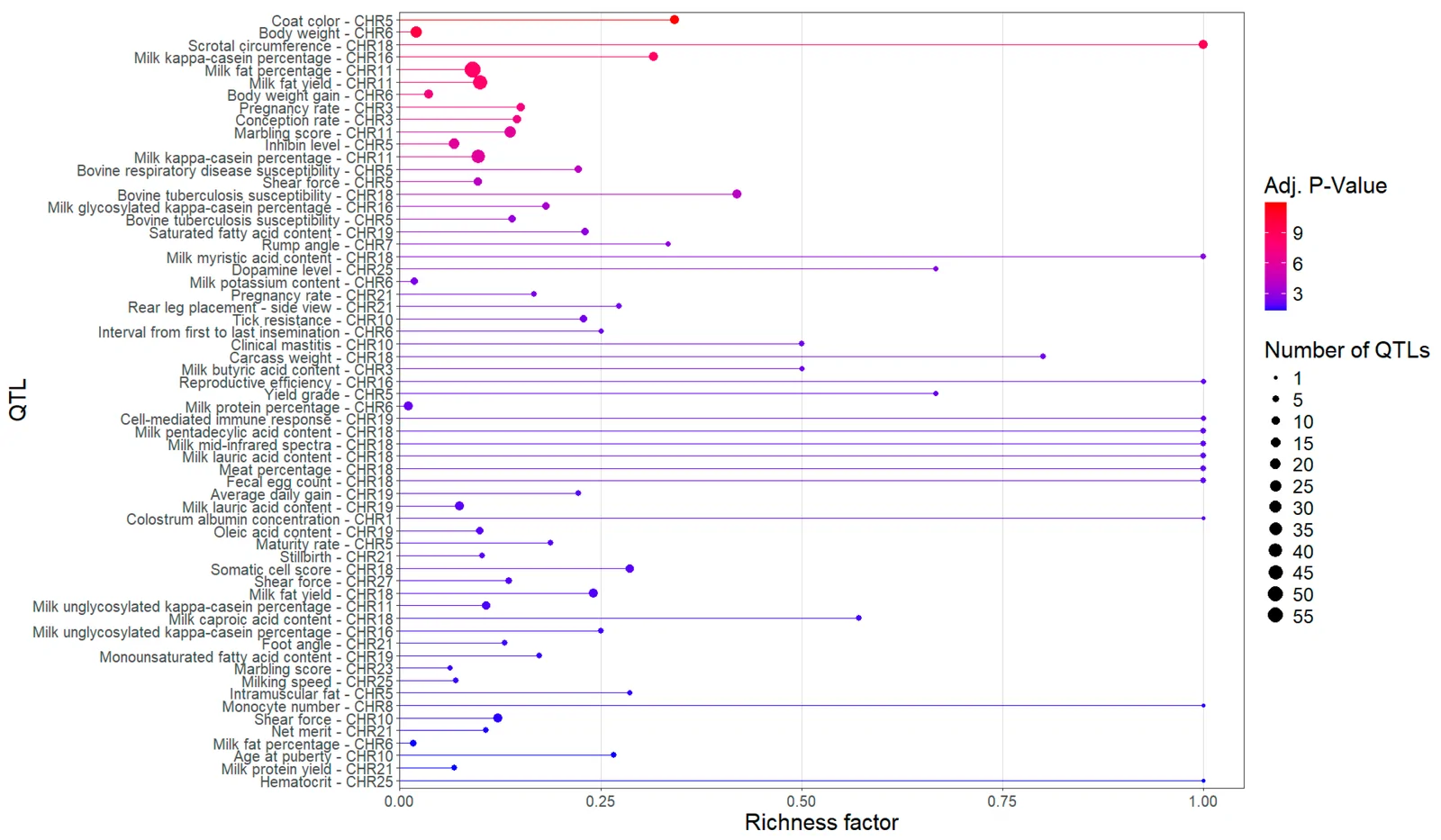
Lollipop plot of individual Quantitative Trait Loci (QTL) significantly enriched within candidate selection signature regions in the North American Corriente cattle population. The x-axis shows the richness factor (proportion of QTL-associated genes overlapping selection signatures relative to total genes annotated to that QTL). Dot size represents the number of QTLs per trait. Dot color indicates the adjusted *p*-value, with red denoting greater significance.

Complementing these findings, a total of 1243 QTLs were identified within the selected regions (Table S4). QTL enrichment analysis revealed that candidate selection signature regions overlapped with previously reported QTLs spanning six functional trait categories, with Milk (37.7%) and Meat and Carcass (18.1%) traits contributing the largest proportions of QTLs, while Production (14.8%), Reproduction (12.8%), Exterior (9.1%), and Health (7.5%) contributed smaller proportions. At the individual QTL level, high richness factors and strong statistical significance were observed for reproduction- and conformation-related traits, including scrotal circumference, body weight, and milk kappa-casein percentage, along with numerous milk fatty acid composition traits, carcass weight, and cell-mediated immune response, many of which mapped to chromosome 18 (Figure 6).

## 3. Discussion

Corriente cattle are one of many Criollo breeds that have evolved and adapted for over five centuries in the Americas, thriving in a wide variety of environments. Despite the wealth of practical knowledge surrounding the breed and their cultural and economic importance, genomic analyses of Corriente cattle in North America remain limited. This study characterized two commercial NAC herds in New Mexico, United States, using a medium-density SNP microarray to explore their population structure, genetic diversity, admixture, and signatures of selection.

Principal Component Analysis revealed that animals from the Roy herd, managed as a closed herd, grouped into a single cluster along with several individuals from the Tucumcari herd. These Tucumcari individuals may be descendants of sires introduced from the Roy herd in 1995. Furthermore, DAPC identified three clusters, suggesting the presence of three putative genetic clusters within the sampled NAC individuals. However, given that the sampling design was limited to two herds, the observed clustering could also reflect local herd structure, founder effects, or management practices rather than true genetically divergent subpopulations. Thus, these findings should be regarded as preliminary and confirmed through more comprehensive sampling covering greater number of NAC herds. The identification of these putative genetic clusters could nonetheless be informative, as distinct bloodlines or herd lineages may serve as distinct reservoirs of genetic material, helping to maintain genetic variation within the breed [14].

Due to the limited sample size and uneven distribution of NAC animals across clusters, individual analysis of the three clusters was not performed. Simply aggregating all individuals into a single dataset could inflate or mask estimates of genetic diversity, linkage disequilibrium structure, and selection signatures. Therefore, all subsequent analyses were conducted using Cluster 3 (*n* = 52) alone, which was considered to have a sufficient sample size for independent analysis.

The heterozygosity values (~0.35) in the NAC population were comparable to those reported for other Criollo cattle populations [8,15,16,17,18,19,20]. However, the NAC population in this study showed higher heterozygosity than the value of 0.27 previously reported for five Mexican Corriente individuals [8]. This difference may be partially attributed to the small sample size in the prior study, which focused on the analysis of multiple cattle breeds globally rather than a single breed as in the present study.

The NAC population presented a moderate-to-high inbreeding level of 14%, higher than that observed for other Criollo cattle breeds [15,18,19,20]. This could be partially explained by the more intensive selection pressure on NAC cattle for conformation, temperament, and mobility traits desired for rodeo sports or improved gait. The high proportion of short ROH segments (<2 Mb) suggests that inbreeding predominantly originated from distant ancestors (20–50 generations ago). Conversely, the low proportion of long ROH segments (>16 Mb) indicates a low level of recent inbreeding originating from recent ancestors (approximately three generations ago) [21]. The ROH length distribution may indicate controlled consanguineous matings in the NAC population, potentially mitigating the negative effects of recent inbreeding on fitness and performance [22,23].

Genetic diversity of NAC was also assessed by estimating the historical Ne. The most recent Ne estimate for NAC was 76, which is above the FAO [24] threshold of 50 for an endangered population but below the recommended Ne of 100 [25]. Other Criollo cattle populations also show reduced Ne [15,16,19,20,26,27], consistent with an overall decline in Criollo cattle populations, primarily attributed to the prevalence of specialized commercial cattle breeds introduced in the Americas [16].

Historical records suggest Corriente cattle in North America were nearly extinct by the late 1800 s due to replacement by European and Indicine cattle [1,11]. Interestingly, this historical account may be temporally consistent with a drastic decline in Ne observed in this study 18–19 generations ago (~1929–1934; 90–95 years ago, assuming a five-year generation interval). However, the Ne trajectories reflect genetic patterns rather than direct historical evidence, and this correspondence should be interpreted as a hypothesis rather than a confirmed causal link. The steep decline continued until 11–12 generations ago (~1964–1969, 55–60 years ago), after which it stabilized. This pattern may tentatively coincide with the subsequent reintroduction of Mexican Criollo cattle for rodeo sport in the mid-1900 s and the later establishment of NACA in 1982. Given the scarcity of historical records documenting the lineage of the Corriente foundational stock [10], the present findings may offer a tentative, hypothesis-generating framework for understanding the ancestry, history, and development of Corriente cattle in North America.

Overall, genetic diversity analyses indicate moderate-to-high genetic diversity in the NAC population. Furthermore, the historical trajectory of the Ne suggests that existing mating plans have been successful in preventing severe inbreeding and loss of genetic variability in recent generations. Since analyses were performed only on Cluster 3, broader sampling from each identified cluster is needed to better characterize the current state of the NAC in northeastern New Mexico, United States.

The combination of DAPC and ADMIXTURE provided insights into the genetic structure of the NAC population and the apparent relationship with taurine and Indicine breeds. The DAPC may reflect evolutionary divergence between Criollo cattle adapted to hot, dry climates (NAC, RCJER, RCRET, and TXL) and those adapted to hot, humid conditions (CRK, CCC, RMS, and SNM). The proximity of NAC with RCJER, RCRET, and TXL in the DAPC may suggest shared genetic structure and ancestry among these breeds, which appear broadly consistent with their geographic proximity [6,9]. Previous studies have similarly reported a close genetic relationship between NAC, RC, TXL, and other Mexican Criollo cattle populations [6,7,8,9,15,28]. This apparent genetic relationship may be consistent with the historical dispersal of Criollo cattle, which entered North America through the Mexican port of Veracruz before expanding into the southwestern United States [29].

North American Corriente cattle showed evidence of substantial Iberian ancestry, consistent with patterns reported in other Criollo breeds [7,15,16,30,31]. A close genetic relationship among NAC, RC, and TXL was also suggested by the ADMIXTURE analysis, where these breeds displayed a similar structure at K = 2–5. At K = 6, NAC exhibited a slightly different admixture pattern, and by K = 7 it appeared more distinct from other Criollo populations. This may support the notion that Criollo breeds are generally distinct from one another [6,7,9]. McTavish et al. [8] reported ~10% of Indicine introgression at K = 2 in five Mexican Corriente cattle, whereas the selected NAC individuals in this study showed ~4% at K = 2. Similarly, comparatively low levels of Indicine introgression have been reported in TXL (~8–11%; [8,16]) and RC cattle (~7–10%; [15]), although direct comparisons across studies should account for differences in reference panels and analytical approaches.

Genomic regions showing evidence of positive selection in NAC cattle were identified using three complementary statistical methods—Tajima’s D, ROH, and iHS—integrated within the DCMS framework. This approach accounts for correlations between univariate statistics, enabling precise and unbiased detection of selected regions [32].

The convergence of functional enrichment and QTLs related to muscle development, cardiac output, and heart rate regulation might be broadly consistent with the athletic phenotype characteristic of NAC cattle, which have reportedly been selected for rodeo and roping sports, as well as for adaptation to arid rangeland conditions [11,12]. Selection acting on genes associated with cardiovascular and skeletal muscle function could potentially reflect adaptation to these sports and improved gait, although this interpretation remains speculative. This pattern may be comparable to selection signatures previously reported in Criollo cattle from Guadalupe, historically used for traction and cart race competition [33], and in other athletic or working animal populations (e.g., racing horses and sled dogs) [34,35]. Additionally, the identification of QTLs for body weight, carcass weight, marbling score, and shear force, together with functional enrichment for muscle development and contraction pathways, is consistent with the possibility that selection in NAC may have shaped genomic regions relevant to growth, lean meat, and carcass composition, although further studies would be needed to confirm this.

The enrichment of immune-related processes (e.g., granulocyte/macrophage differentiation, complement activation, innate immune response, and cytokine regulation) could potentially reflect selective pressures associated with harsh natural environments, pathogen exposure, and minimal human-mediated health management, which may be broadly consistent with the reported hardiness and disease resistance attributed to Criollo cattle [10,12].

Several reproduction-related QTLs (e.g., scrotal circumference, pregnancy rate, conception rate) overlapped with the identified selection signatures. Although such overlap does not demonstrate that selection specifically targeted fertility, it is consistent with the reported favorable reproductive performance of Criollo cattle under extensive management systems [10,12]. This finding warrants further investigation.

Although Corriente cattle are not conventionally selected for high milk yield and commercial milk production, the identification of milk-associated QTLs may be explained by a pleiotropic genomic architecture shared with other physiological pathways, or by a retained ancestral signal from the foundational breed. Alternatively, this finding may be related to limitations inherent to the QTL database annotations, which are predominantly derived from commercial dairy and beef populations (e.g., Holstein and Angus; [36]) and may not fully capture trait associations relevant to less-studied breeds like Corriente and other Criollo breeds.

Together, these results are consistent with the possibility that selection in NAC may have shaped genomic regions underlying physical, reproductive, and immune-related traits, which broadly aligns with the natural selection history of Criollo cattle, distinct from commercial beef or dairy cattle breeds. Previous studies have also detected candidate regions in Criollo cattle breeds linked to several molecular processes underlying heat tolerance, adaptation to poor-quality forage conditions, DNA repair processes, immune response, and adaptation of male and female reproductive functions under resource-limiting conditions [16,33,37]. Given the anecdotic and practical knowledge of Corriente cattle and the identification of genes and QTLs related to relevant traits in the present study, follow-up functional studies are warranted to disentangle and clarify the functional relevance of these overlapping genomic regions in the NAC population.

## 4. Materials and Methods

### 4.1. Animals and Samples

Animal handling and sampling protocols were approved by the New Mexico State University Institutional Animal Care and Use Committee (protocol #2312000907, approved on 20 February 2024). A total of 97 ear tissue samples (Tissue Sampling Unit, Allflex, Madison, NJ, USA) from NAC cattle were obtained at two commercial ranches in northeastern New Mexico, United States: one located in Roy, Harding County (*n* = 47), and the other in Tucumcari, Quay County (*n* = 50). The Roy herd samples consisted of 27 adults (24 females and 3 males) and 20 yearlings (11 females and 9 males), while the Tucumcari herd samples consisted of 50 nursing calves approximately 3–4 months of age (25 females and 25 males). Sampling of the Tucumcari herd was limited to nursing calves, as facility infrastructure and herd management practices at the time restricted access to other age classes.

The two herds sampled were chosen as they represent typical NAC commercial populations selected primarily for sporting purposes (e.g., roping and bulldogging) and beef production. The primary beef production strategy involves commercializing purebred NAC females for crossbreeding with sires from specialized beef cattle breeds. The Roy herd, established in 1977, is a closed herd of approximately 70 dams and is registered with the NACA. In contrast, the Tucumcari herd, established in 1992, comprises about 1200 dams and was registered with the NACA until shortly before sampling for this study. The Tucumcari herd introduced sires from various NAC breeders, including the Roy herd on one occasion in 1995.

### 4.2. Genotyping and Quality Control

Genotyping was performed using the VM2 SNP array (63,683 SNPs; Genetic Visions-STTM, Middleton, WI, USA), which is mapped to the bovine genome assembly ARS-UCD1.2/bosTau9. Data from the two herds were merged, and quality control was conducted using PLINK v.1.9 [38], following the FAO’s bioinformatics pipeline for genomic characterization of animal genetic resources [39]. SNPs with missing chromosome or position information, along with those located on sex chromosomes, were excluded prior to quality control filtering using the *--autosome* flag in PLINK v1.9, which restricts the dataset to autosomal SNPs before subsequent filters are applied (61,283 SNPs remaining). Quality control parameters included individual missingness (*--mind < 0.05;* 61,283 SNPs remaining), missing call rate per SNP (*--geno < 0.05;* 58,847 SNPs remaining), deviation from Hardy–Weinberg equilibrium (*--hwe 0.000001;* 58,837 SNPs remaining)—a stringent threshold selected to remove SNPs with likely genotyping errors while retaining those deviating due to genuine population structure—and minor allele frequency (*--maf > 0.01; 56,604* SNPs remaining).

Relatedness was estimated using the KING robust kinship method [40] in PLINK v2.0 [41]. One individual from each pair with a kinship coefficient > 0.177 (indicative of a parent-offspring or full-sibling relationship) was removed from the dataset.

### 4.3. Genetic Structure of North American Corriente Cattle Population

Principal Component Analysis was performed using PLINK v1.9 and visualized with the R package ggplot2 v3.5.0 [42]. The scatterplot revealed that animals from the Tucumcari herd intermingled with those from the Roy herd (Figure 1a), prompting further investigation of genetic structure using DAPC.

Unlike PCA, which summarizes overall genetic variability, DAPC specifically maximizes between-group diversity while minimizing within-group diversity [43]. Discriminant Analysis of Principal Components is particularly effective for identifying and describing clusters of genetically related individuals, making it well-suited for elucidating complex population structures [43], as appears to be the case for the NAC herds analyzed in this study. The DAPC analysis was conducted using the R package adegenet v2.1.10 [44]. The *find.clusters* function was used to determine the optimal number of clusters (K) from a range of 1 to 15, with selection based on the BIC value. The optimal number of Principal Components (PC) to retain was determined through the cross-validation procedure using the *xvalDapc* function.

Discriminant Analysis of Principal Components identified three distinct clusters, designated as Cluster 1 (*n* = 10), 2 (*n* = 18), and 3 (*n* = 52) (Figure 1b). Because sample sizes varied considerably across clusters, and Cluster 1 and 2 were too small to support reliable individual analysis, all individuals were not pooled into a single dataset because doing so risked distorting estimates of genetic diversity, linkage disequilibrium structure, and selection signatures. Cluster 3 was therefore selected for downstream analyses, as it was the only cluster with a sample size considered adequate for independent evaluation.

### 4.4. Genetic Diversity of North American Corriente Cattle Population

Genetic diversity was evaluated through four different parameters: H_O_, H_E_, F_ROH_, and Ne. The H_O_ and H_E_ were computed using PLINK v1.9. ROH were identified with the consecutive runs method [45] implemented in the R package detectRUNS v0.9.6 [46]. The following parameters were used: (i) maximum gap between consecutive homozygous SNPs: 1000 kb; (ii) minimum ROH length: 250 kb; (iii) maximum number of opposite genotypes: 1; (iv) maximum number of missing genotypes: 1; and (v) minimum number of SNPs in a ROH: 23, calculated using the formula proposed by Lencz et al. [47] and modified by Purfield et al. [48]. The number of ROH was estimated per individual and classified into five length categories: >0–2 Mb, >2–4 Mb, >4–8 Mb, >8–16 Mb, and >16 Mb. Additionally, FROH for each animal was calculated.

Effective population size was estimated using GONE software v29/08/2021 [49], following default software recommendations: unknown phase (*PHASE = 2*), an assumed recombination rate of 1 cM/Mb (*cMMb = 1*) with Haldane’s correction (*DIST = 1*), no MAF filter (MAF = 0.0), retention of zero-value SNPs (*ZERO = 1*), all autosomes (*maxNCHROM = −99*), up to 50,000 SNPs per chromosome (*maxNSNP = 50,000*), and a maximum recombination fraction of 0.05 (*hc = 0.05*). LD data span 2000 generations in 400 bins of 5 generations each (*NGEN = 2000*, *NBIN = 400*), with Ne estimated as the geometric mean across 40 replicates (*REPS = 40*), following default software recommendations. The results were plotted using the R package ggplot2 v3.5.0.

### 4.5. Genetic Admixture in the North American Corriente Cattle Population and Their Relationship to Other Cattle Breeds

The admixture in NAC and its relationship with Criollo and other cattle breeds were explored using DAPC and ADMIXTURE. The NAC population data were merged with datasets from six previously characterized Criollo cattle breeds [16]: CCC, RMS, SNM, CRK, SNP, and TXL. The dataset also included RCJER in New Mexico, United States [16], and RC from Rancho Experimental Teseachi in Chihuahua, Mexico. The RCRET animals were genotyped at the Genetic Visions-ST^TM^ laboratory (Middleton, WI, USA) using the VM2 ~64 K SNP array, and the data were provided by the Universidad Autónoma de Chihuahua, Mexico. This complete merged dataset was also used in a companion study characterizing the ancestry and admixture of a Rarámuri Criollo population in New Mexico, United States [15].

To minimize sample-size bias, a maximum of 15 individuals per Criollo population was selected using the random sampling procedure implemented in the R package BITE v2.1.2 [50], which selects a subset that best preserves the genetic structure of the original dataset based on multidimensional scaling of pairwise identity-by-state distances, validated via a two-sample Kolmogorov–Smirnov test. The final dataset consisted of 105 individuals and 10,277 common SNPs (Table S1).

Discriminant Analysis of Principal Components was performed as described in Section 4.3. The optimal value of K was selected from a range of 1 to 20.

The relative contributions of taurine and Indicine ancestry to the NAC population structure were investigated using the ADMIXTURE v1.4 software [51]. The Criollo cattle dataset was merged with four reference groups from Pitt et al. [16]: (i) Iberian taurine, (ii) commercial taurine, (iii) African taurine, and (iv) Indicine breeds. To minimize sample-size bias, a maximum of 15 individuals per breed was selected using the R package BITE v2.1.2, resulting in a final dataset of 345 individuals and 10,277 common SNPs (Table S1). Admixture analyses were performed from K = 2 to 20, and the optimal K was determined using the fivefold cross-validation procedure in ADMIXTURE v1.4. Results were visualized using the R package BITE v2.1.2.

### 4.6. Selection Signature Analysis in North American Corriente Cattle Population

Three complementary intra-population statistics were used to detect selection signatures in the NAC population: Tajima’s D (based on site frequency), ROH (based on reduced local variability), and iHS (based on linkage disequilibrium).

Tajima’s D values [52] were calculated in non-overlapping 300 kb sliding windows using VCFtools v0.1.16 [53] and assigned to SNPs within each window; missing values were set to zero to represent a neutral, “no signal” expectation and preventing missing data from being falsely flagged as extreme during DCMS integration. ROH were identified with the consecutive runs method using the R package detectRUNS v0.9.6, as described in Section 2.4. iHS [54] was computed using the R package rehh v3.2.2 [55]; absolute iHS values were used and missing values were set to zero. Prior to analysis, genotype phasing was performed using Beagle v5.4 software with default settings [56]. The three statistics were integrated using the DCMS framework [32] to account for inter-statistic correlations, enhancing resolution and reducing false-positive rates.

The DCMS analysis was performed using the R package MINOTAUR v0.0.1 [57], following established methodologies [58,59]. The workflow consisted of the following steps: first, fractional ranks of ROH incidence and Tajima’s D, together with absolute iHS values, were used to calculate *p*-values for each statistic using the *stat_to_pvalue*. Tail direction reflected the expected deviation under positive selection: Tajima’s D was tested left-tailed, as selective sweeps skew D toward negative values [52], while ROH and iHS were tested right-tailed, since extended ROH reflects reduced haplotype diversity from hitchhiking [48], and absolute iHS values consolidate selection signals from either allele into a single right tail [54]. This approach is consistent with established DCMS applications [32,58,59]. Next, a robust covariance matrix between statistics was estimated using the *covMcd* function (*alpha = 0.75, nsamp = 50,000*) from the R package robustbase v0.99-4-1 [60]. This matrix was used to compute DCMS scores, which were then fitted to a normal distribution via robust linear modeling with the *rlm* function from the R package MASS v7.3-65 [61]. The fitted DCMS values were converted to *p*-values using the *pnorm* R function. Finally, *p*-values were adjusted for multiple testing using the Benjamini–Hochberg false discovery rate correction [62] via the *p.adjust* R function.

SNPs with adjusted *p*-values (q-values) < 0.05 were considered significant. Results were visualized in a Manhattan plot generated with the R package qqman v0.1.9 [63].

### 4.7. Candidate Genes and Quantitative Trait Loci in North American Corriente Cattle Population

Putative regions under selection were defined as those extending 250 kb upstream and downstream of each significant SNPs [64,65]. Gene and QTL annotations were performed using the R package GALLO v1.5 [66]. The gene annotation file was obtained from Ensembl (ARS-UCD1.2 release 110; [67]), and the QTL annotation file was obtained from the Animal Genome cattle QTL database (release 56; [36]).

Genes were functionally annotated by identifying significant (*p*-value < 0.05) GO and KEGG terms using the Database for Annotation, Visualization, and Integrated Discovery (DAVID) v2026_1 [68]. The analyses allowed the identification of molecular functions, biological processes, and pathways for the genes included in the putative regions under selection. QTL enrichment analysis was conducted using the R package GALLO v1.5, with a false discovery rate threshold of <0.05 considered statistically significant.

## 5. Conclusions

The present study was motivated by the limited peer-reviewed scientific research on NAC cattle genomics, despite their status as a threatened breed and the considerable body of anecdotal and practical knowledge about the breed. The results suggest that NAC cattle could represent a divergent Criollo cattle breed, although this hypothesis would need to be confirmed by additional sampling of individuals across multiple independent and geographically representative herds. Functional and QTL enrichment analyses identified genomic regions potentially related to athletic performance, fertility, immune competence, and milk and carcass traits, which may be consistent in part with the action of natural selection; however, these findings are preliminary and would require further validation through functional studies before firm conclusions can be drawn. Overall, this study may contribute to ongoing efforts to genetically characterize, promote, and conserve Criollo cattle populations across the Americas.

## Data Availability

The dataset is not publicly available due to private ownership of the animals from which samples were collected. Nevertheless, the data can be shared upon request to the corresponding authors.

## Supplementary Materials

The following supporting information can be downloaded at: https://www.mdpi.com/article/10.3390/ijms27187982/s1.

## Abbreviations

The following abbreviations are used in this manuscript:

ANG	Angus
BAO	Baoule
BEC	Berrenda en Colorado
BIC	Bayesian Information Criterion
BRM	Brahman
CAC	Cachena
CAR	Cardena Andaluza
CCC	Costeño con Cuernos
CRK	Florida Cracker
DA	Discriminant Axis
DAPC	Discriminant Analysis of Principal Components
DCMS	Decorrelated Composite of Multiple Signals
F_ROH_	Inbreeding coefficient based on runs of homozygosity
GIR	Gir
GO	Gene Ontology
H_E_	Expected heterozygosity
H_O_	Observed heterozygosity
HOL	Holstein
iHS	Integrated Haplotype Score
JER	Jersey
KEGG	Kyoto Encyclopedia of Genes and Genomes
LAG	Lagune
LID	Lidia
MOS	Mostrenca
MSH	Milking Shorthorn
NAC	North American Corriente
NACA	North American Corriente Association
NDA	N’Dama
Ne	Effective population size
NEL	Nelore
PAJ	Pajuna
PCs	Principal components
PCA	Principal component analysis
QTL	Quantitative Trait Loci
RAV	Asturiana de los Valles
RC	Rarámuri Criollo
RCJER	Rarámuri Criollo from the Jornada Experimental Range
RCRET	Rarámuri Criollo from the Rancho Experimental Teseachi
RET	Retinta
RMS	Romosinuano
ROH	Runs of homozygosity
RPO	Red Poll
SEN	Senepol
SHR	Beef Shorthorn
SNM	San Martinero
SNP	Single Nucleotide Polymorphism
SOM	Somba
TXL	Texas Longhorn
