# Supplementary material for: Genetic Diversity and Selection Signatures of Two North American Corriente Cattle Herds in Northeastern New Mexico, United States
Source: Int J Mol Sci. 2026 Sep 8;27(18):7982. doi: 10.3390/ijms27187982 (PMC13606608; doi:10.3390/ijms27187982)
Supplement: Supplementary file 1 [file ijms-27-07982-s001.zip › Figures S1-S5.pdf]

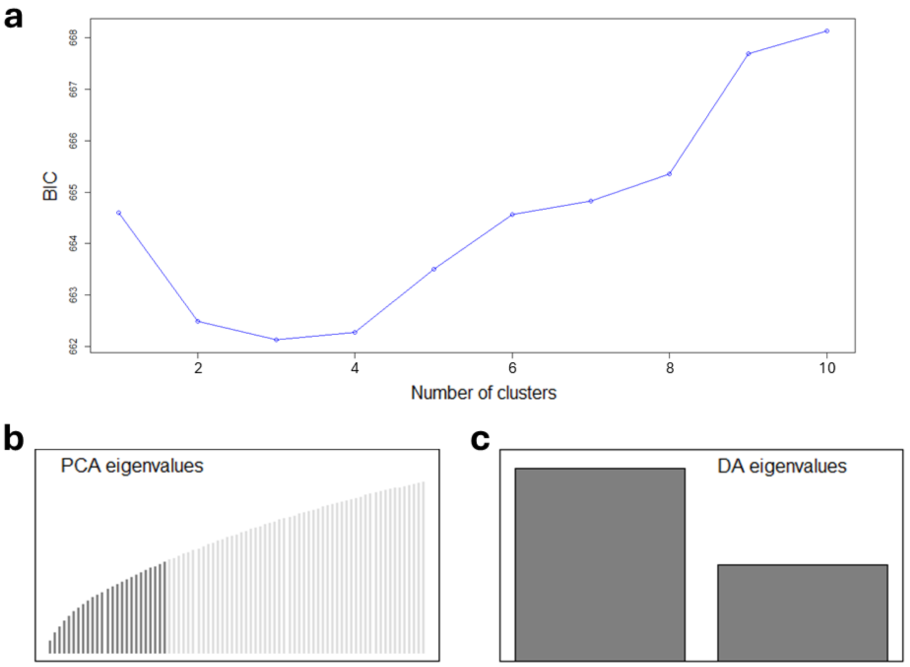

Figure S1. Discriminant Analysis of Principal Component of North American Corriente cattle herds. (**a**) Inference of the number of clusters based on K-means algorithm. (**b**) twenty-five Principal Components (PC) and (**c**) two Discriminant Axes (DA) were retained.

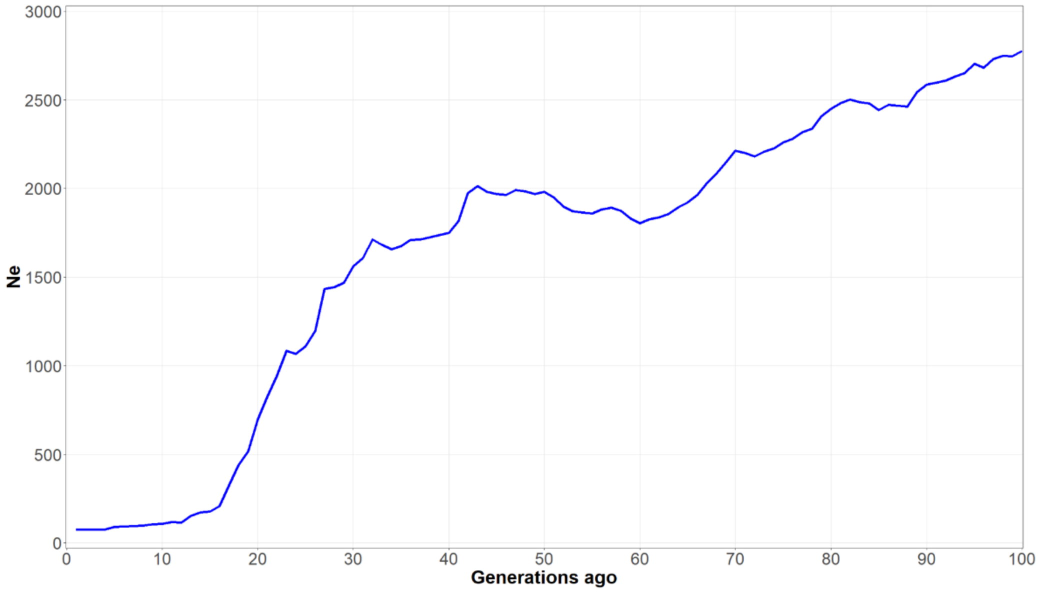

Figure S2. Historical effective population size of North American Corriente cattle population calculated using the GONE software program.

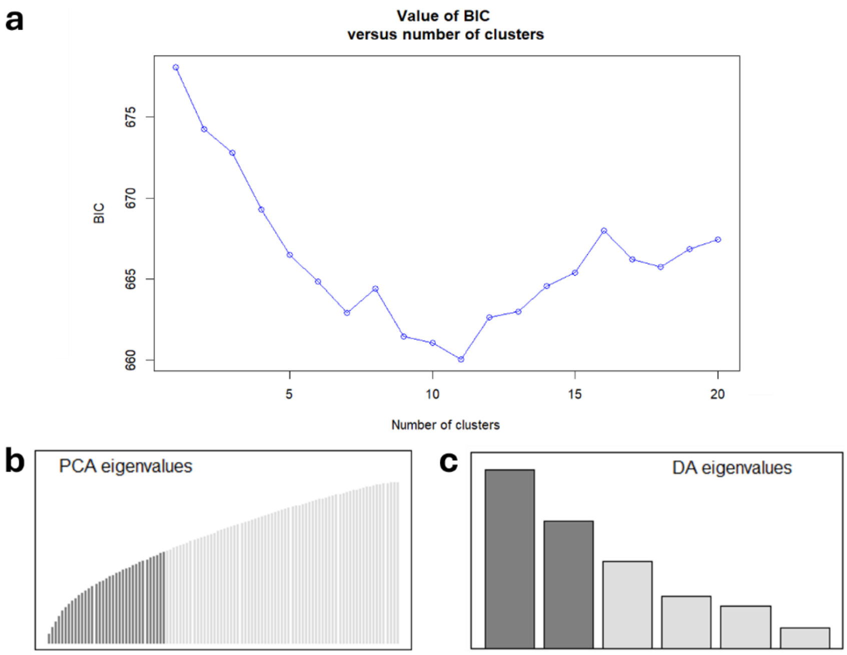

Figure S3. Discriminant Analysis of Principal Component of Criollo cattle populations. (**a**) Inference of the number of clusters based on K-means algorithm, (**b**) thirty-five Principal Components (PC), and (**c**) six Discriminant Axes (DA) were retained.

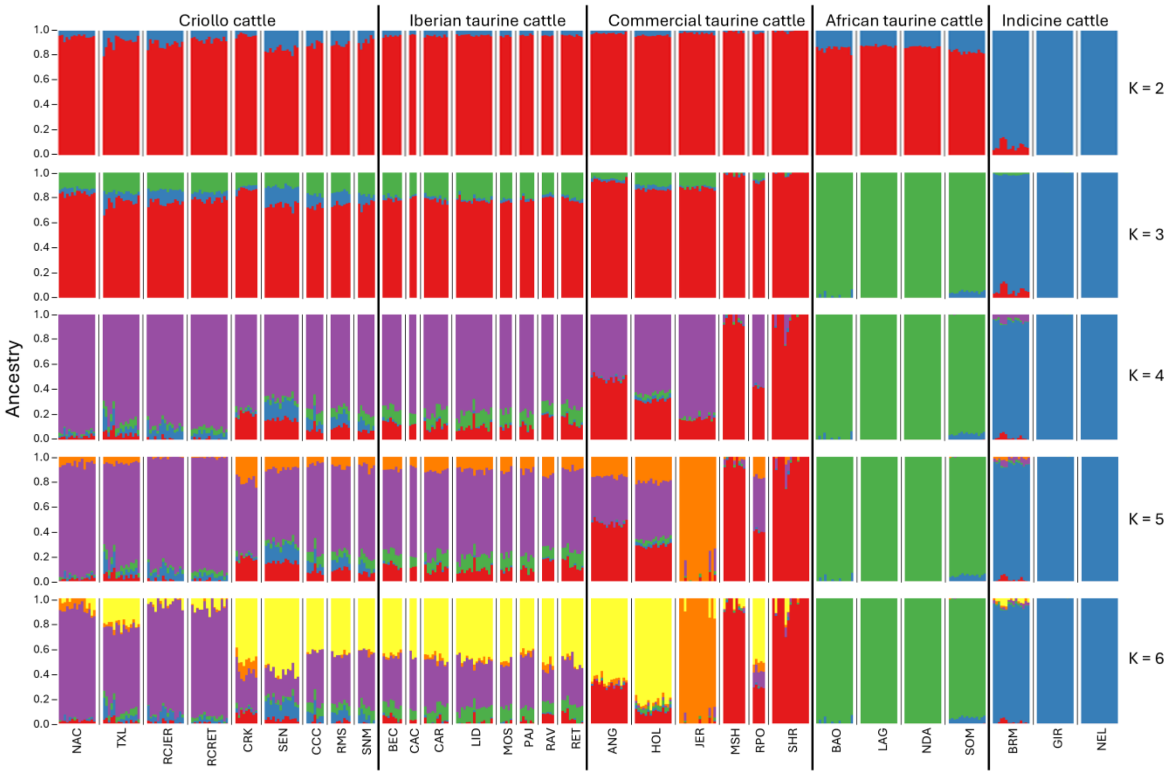

Figure S4. Admixture analysis of North American Corriente cattle (K = 2 – K = 20) along with Criollo, taurine, and Indicine cattle breeds. NAC: North American Corriente, TXL: Texas Longhorn, RCJER: Rarámuri Criollo cattle from Jornada Experimental Range, RCRET: Rarámuri Criollo cattle from Rancho Experimental Teseachi, CRK: Florida Cracker, SNP: Senepol, CCC: Costeño con Cuernos, RMS:

Romosinuano, SNM: San Martinero, BEC: Berrenda en Colorado, CAC: Cachena, CAR: Cardena Andaluza, LID: Lidia, MOS: Mostrenca, PAJ: Pajuna, RAV: Asturiana de los Valles, RET: Retinta, ANG: Angus, HOL: Holstein, JER: Jersey, MSH: Milking Shorthorn, RPO: Red Poll, SHR: Beef Shorthorn, BAO: Baoule, LAG: Lagune, NDA: N'Dama, SOM: Somba, BRM: Brahman, GIR: Gir, NEL: Nelore.

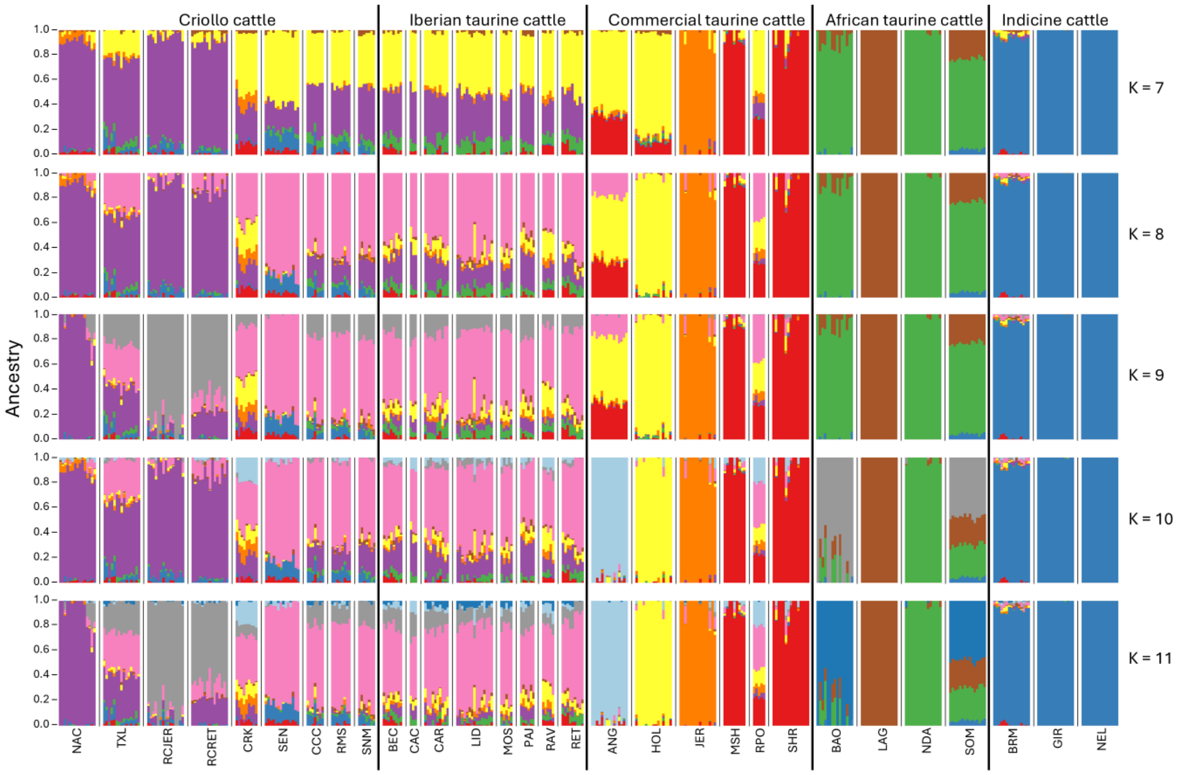

Figure S4 (continued). Admixture analysis of North American Corriente cattle (K = 2 – K = 20) along with Criollo, taurine, and Indicine cattle breeds.

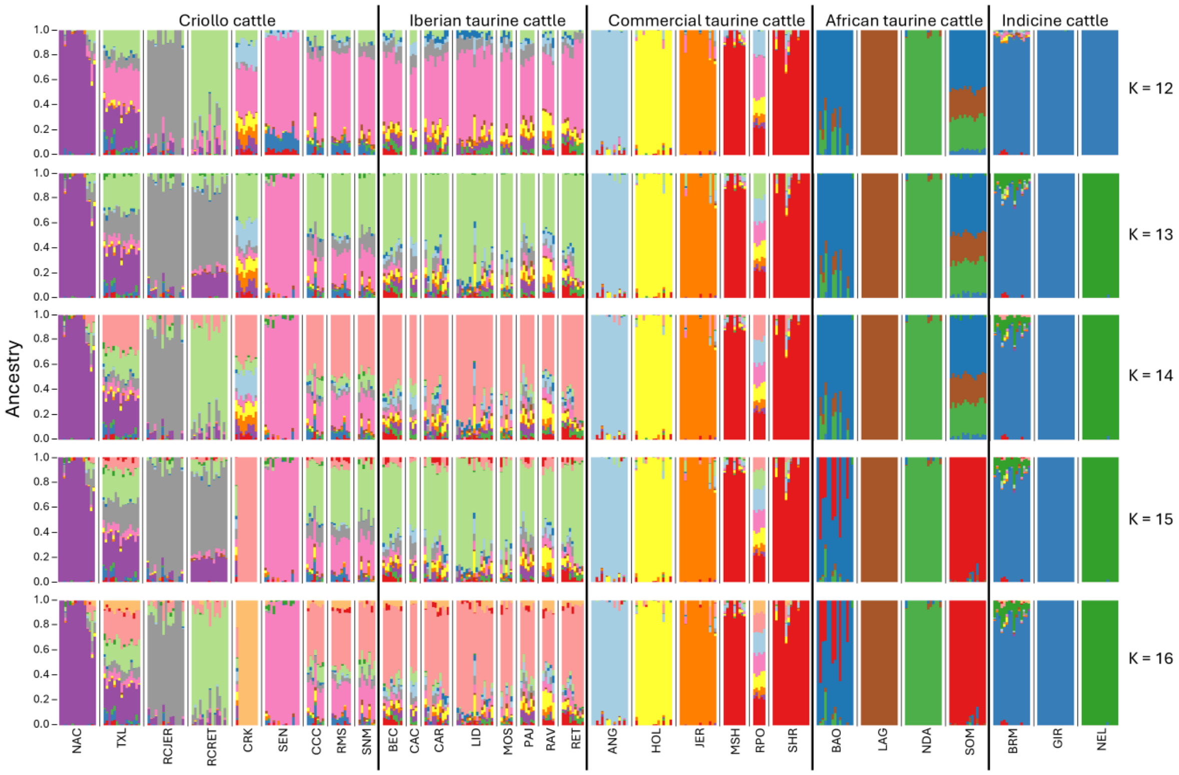

Figure S4 (continued). Admixture analysis of North American Corriente cattle (K = 2 – K = 20) along with Criollo, taurine, and Indicine cattle breeds.

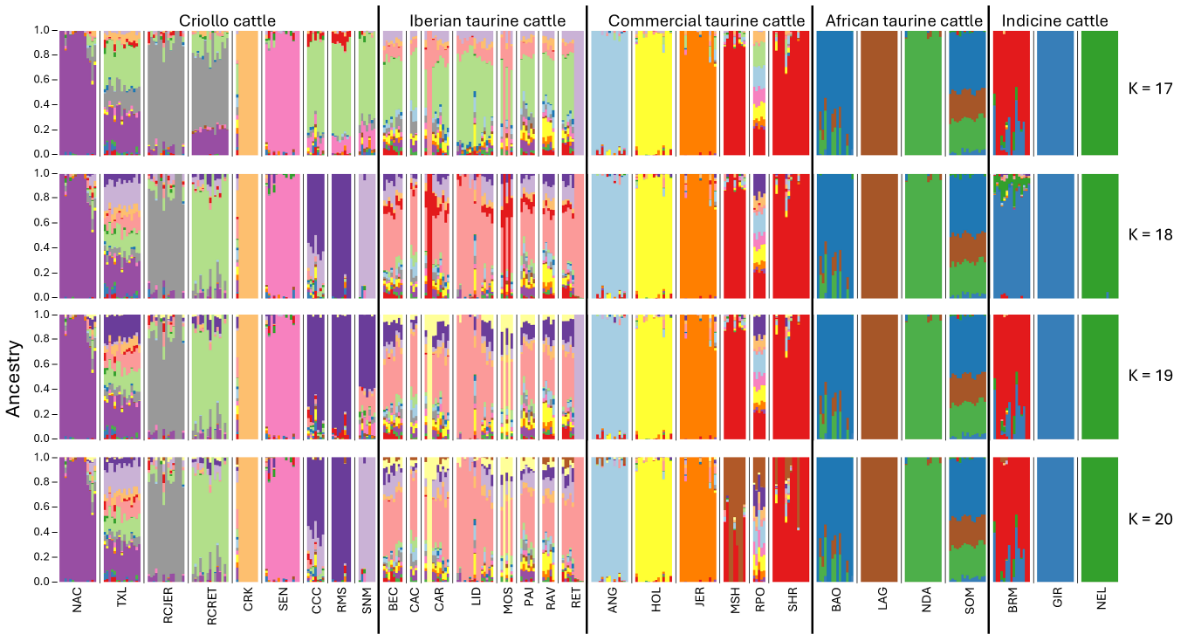

Figure S4 (continued). Admixture analysis of North American Corriente cattle (K = 2 – K = 20) along with Criollo, taurine, and Indicine cattle breeds.

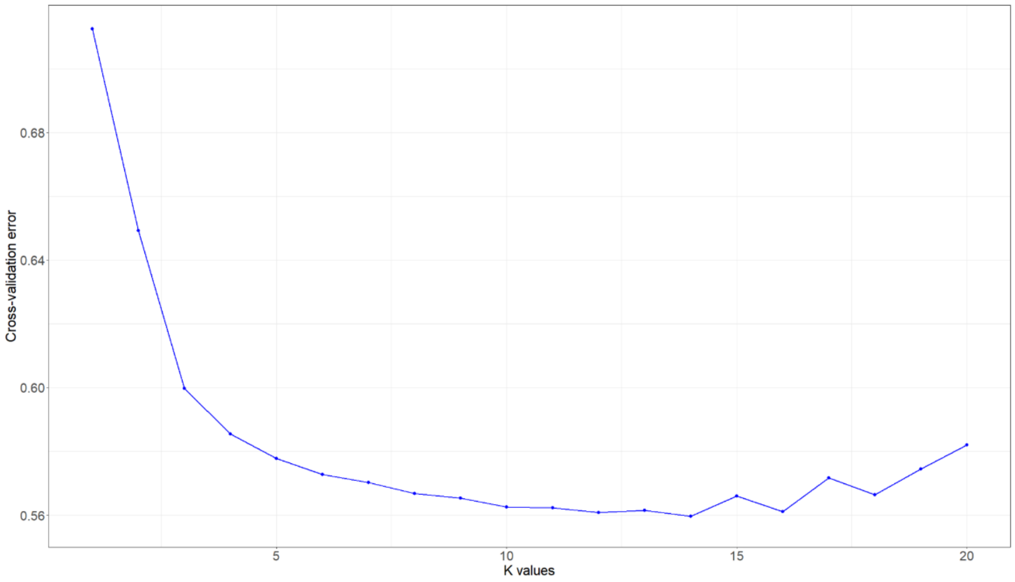

Figure S5. Cross-validation error in the admixture analysis for clusters (K) varying from 2-20.
